# Porcine Epidemic Diarrhea-Virus-Induced Cell Death: Mechanistic Insights and Therapeutic Strategies

**DOI:** 10.3390/vetsci12111050

**Published:** 2025-11-01

**Authors:** Miao Zhang, Maoyuan Yin, Penggang Liu

**Affiliations:** 1 College of Veterinary Medicine, Yangzhou University, Yangzhou 225009, China; dx120220186@stu.yzu.edu.cn; 2 International Joint Research Laboratory in Universities of Jiangsu Province of China for Domestic Animal Germplasm Resources and Genetic Improvement, Yangzhou University, Yangzhou 225009, China; 3 Heze Vocational College, Heze 274002, China; yinmaoyuan@hezevc.edu.cn

**Keywords:** porcine epidemic diarrhea virus, apoptosis, necroptosis, pyroptosis, PANoptosis, treatment

## Abstract

Porcine epidemic diarrhea virus (PEDV), a member of the family Coronaviridae, markedly increases morbidity and mortality in neonatal piglets, posing a significant threat to the global swine industry. Cell death represents a double-edged sword: it eliminates infected cells and helps maintain host homeostasis, but it contributes to disease development and progression when dysregulated. Numerous coronaviruses have been shown to induce cell death through well-characterized mechanisms; however, the interplay between PEDV and host cell death remains insufficiently defined. This review seeks to comprehensively delineate the molecular mechanisms by which PEDV modulates cell death, focusing on apoptosis, necroptosis, pyroptosis, and their integrated form, PANoptosis. Furthermore, we summarize current therapeutic strategies targeting these pathways, providing a theoretical framework and potential directions for developing interventions against PEDV infection.

## Simple Summary

Porcine epidemic diarrhea virus (PEDV) regulates host cell death through multiple signaling pathways, influencing both the intensity of host immune responses and the extent of intestinal injury. This review focuses on the mechanisms by which PEDV regulates apoptosis, necroptosis, pyroptosis, and their integrated form, PANoptosis. Additionally, elucidating the dual role of cell death in facilitating viral clearance while exacerbating tissue injury provides a theoretical framework and potential directions for developing targeted antiviral therapies.

## 1. Introduction

Porcine epidemic diarrhea virus (PEDV), the causative agent of porcine epidemic diarrhea, is an enveloped, positive-sense, single-stranded RNA virus belonging to the genus Alphacoronavirus, family Coronaviridae, order Nidovirales. PEDV can infect pigs of all ages, but piglets are especially susceptible, with mortality rates reaching 90–100% in suckling piglets less than three days old. Clinically, the disease is characterized by acute watery diarrhea, vomiting, and dehydration, often accompanied by anorexia and lethargy. Pathologically, PEDV primarily targets villous epithelial cells of the small intestine, causing villous atrophy, disruption of tight junctions, decreased mucin secretion, and reduced digestive enzyme activity, which together result in malabsorption, dehydration, and high mortality [1,2,3]. Since its initial emergence in the United Kingdom and Belgium in the late 1970s, PEDV has caused outbreaks, most notably the devastating epidemic in the United States in 2013, which resulted in a loss of approximately 10% of the national pig population [4]. Epidemiological studies indicate that PEDV prevalence remains substantial in many swine-producing regions. For example, a study conducted in Shandong Province, China, reported PEDV positivity rates of 34.88% in 2019, 39.33% in 2020, and 36.36% in 2021, indicating a moderate to high prevalence in these areas [5]. More recently, recurrent epidemics in major swine-producing countries across Asia, including China, South Korea, and the Philippines, have highlighted the ongoing threat to global swine health [6]. PEDV not only elevates morbidity and mortality in neonatal piglets but also increases costs associated with vaccination and biosecurity measures, thereby posing a persistent and substantial economic burden on the pig industry worldwide.

PEDV infection causes severe diarrhea in piglets, which is closely associated with intestinal epithelial injury. Increasing evidence indicates that PEDV infection induces cell death in intestinal epithelial cells. These processes lead to villous epithelial shedding, tight junction disruption, and barrier dysfunction, ultimately contributing to malabsorption and diarrhea. Therefore, understanding how PEDV manipulates cell death pathways is essential for elucidating its pathogenic mechanisms and developing effective antiviral strategies.

Over the past decades, more than twenty forms of cell death have been identified, among which classical programmed cell death, such as apoptosis, necroptosis, and pyroptosis, have garnered significant attention. Programmed cell death constitutes a fundamental biological process for maintaining tissue homeostasis and shaping host infection response [7]. Increasing evidence demonstrates that cell death and viral infection are tightly interconnected in a complex and dynamic interplay. On one hand, host cells employ death pathways as an antiviral strategy to eliminate infected cells and restrict viral replication and dissemination. On the other hand, viruses have evolved diverse mechanisms to exploit these pathways to favor their replication and spread. Disruption of this balance often results in severe tissue damage and immune dysregulation, amplifying disease severity [8,9]. Currently, the relationship between PEDV and host cell death remains incompletely understood; however, insights could be drawn from studies of other coronaviruses, including Middle East respiratory syndrome coronavirus (MERS-CoV) and severe acute respiratory syndrome coronavirus 2 (SARS-CoV-2). In this review, we summarize current knowledge of the mechanisms of PEDV-induced cell death and discuss potential therapeutic approaches targeting these pathways, with particular emphasis on drug repurposing and the development of novel antiviral strategies.

## 2. PEDV Infection and Apoptosis

### 2.1. Apoptosis Machinery

Apoptosis is a tightly regulated form of programmed cell death essential for maintaining cellular homeostasis and regulating various physiological and pathological processes. Morphologically, it is characterized by cell shrinkage, chromatin condensation, fragmentation, and the formation of apoptotic bodies [10]. Two major signaling routes govern apoptosis: the intrinsic (mitochondrial) and extrinsic (death-receptor-mediated) pathways.

The intrinsic pathway is typically triggered when mitochondrial outer membrane integrity is compromised, often in response to signals such as reactive oxygen species (ROS) accumulation, DNA damage, endoplasmic reticulum (ER) stress, or viral infection. This process is orchestrated by the BCL-2 protein family, which can be broadly divided into three groups: anti-apoptotic proteins (e.g., BCL-2, BCL-XL, BCL-W, and BCL-B), pro-apoptotic effectors (e.g., BAX, BAK, and BOK), and BH3-only proteins (e.g., BAD, BIK, and BID), which either directly activate BAX/BAK or neutralize anti-apoptotic proteins [11,12,13]. Activated BAX/BAK oligomerize at the mitochondrial outer membrane to form large pores, mediating mitochondrial outer membrane permeabilization (MOMP) and releasing pro-apoptotic factors, including cytochrome c, Smac/DIABLO, and apoptosis-inducing factor (AIF), into the cytosol [13]. Cytochrome c binds to apoptotic protease-activating factor-1 (APAF-1) together with pro-caspase-9 to form the apoptosome, which triggers caspase-9 activation and subsequently drives the cleavage of executioner caspase-3, -6, and -7, ultimately leading to cell death [14,15,16]. In contrast, AIF translocates into the nucleus, which induces chromatin condensation and DNA fragmentation, driving caspase-independent apoptosis [17,18]. Smac/DIABLO promotes apoptosis by binding to inhibitor of apoptosis proteins (IAPs) such as XIAP and c-IAP1/2, thereby releasing their inhibitory constraint on caspases [19].

The extrinsic pathway is initiated when death receptors (DRs) engage their cognate ligands. Major ligand–receptor pairs include FasL (CD95L) with Fas (CD95/APO-1), tumor necrosis factor-α (TNF-α) with TNFR1, and TRAIL with its receptors DR4 (TRAIL-R1) and DR5 (TRAIL-R2) [20,21,22]. Upon ligand binding, adaptor proteins such as Fas-associated death domain protein (FADD) or TNFR1-associated death domain protein (TRADD) are recruited, along with pro-caspase-8 (or pro-caspase-10 in certain cell types), to assemble the death-inducing signaling complex (DISC). Within the DISC, pro-caspase-8 undergoes auto-cleavage to generate active caspase-8, which activates executioner caspases (caspase-3, -6, and -7), culminating in apoptosis [23]. In parallel, caspase-8 can cleave BID to produce truncated BID (tBID), which translocates to mitochondria and promotes BAX/BAK-mediated MOMP, thus linking the extrinsic and intrinsic apoptotic pathways [24].

In addition to the canonical intrinsic and extrinsic pathways, endoplasmic reticulum stress (ERS)-induced apoptosis is often regarded as an upstream branch that intersects with both classical routes through multiple mechanisms. ERS is triggered under protein-folding overload, disruption of Ca^2+^ homeostasis, or oxidative stress. Mild ERS activates the unfolded protein response (UPR) to restore cellular homeostasis, whereas prolonged or excessive stress shifts the UPR toward pro-apoptotic signaling [25]. ERS-induced apoptosis proceeds primarily through three primary mechanisms [26,27]. The first pathway is the PERK–eIF2α–CHOP pathway: PERK activation leads to eIF2α phosphorylation and ATF4/CHOP upregulation [28]. CHOP promotes the transcription of BH3-only proteins such as Bim and PUMA, thereby inducing MOMP and activating the intrinsic pathway, while also upregulating DR5/TRAIL-R2 to amplify death receptor signaling [29]. The second is the IRE1–JNK–BAX axis: IRE1 activates the TRAF2–ASK1–JNK cascade, resulting in BAX/BAK activation, cytochrome c release, and mitochondrial-dependent apoptosis [30]. The third is Ca^2+^ dysregulation: ERS causes Ca^2+^ efflux from the ER, subsequent accumulation in mitochondria, and altered mitochondrial membrane permeability, activating caspase-9/-3–mediated apoptosis.

### 2.2. Apoptosis in PEDV Infection

Increasing evidence indicates that apoptosis plays a critical role in the porcine epidemic diarrhea (PED), contributing to intestinal epithelial cell and tissue damage. Histopathological analyses of PEDV-infected piglets have shown extensive apoptosis in villous enterocytes of the small intestine, confirmed by terminal deoxynucleotidyl-transferase-mediated dUTP nick-end labeling (TUNEL) staining, leading to substantial cell loss. In weaned piglet infection models, PEDV-positive enterocytes exhibited increased expression of caspase-3, accompanied by enhanced markers of ERS and UPR signaling, collectively suggesting the activation of stress responses and apoptosis during infection [31,32].

Extensive evidence has demonstrated that PEDV can induce apoptosis through its viral proteins, thereby facilitating the release and dissemination of progeny virions (Table 1). The spike (S) protein, a major structural glycoprotein, mediates viral attachment and membrane fusion and is divided into two functional subunits, S1 and S2. The S1 subunit binds to host receptors such as aminopeptidase N, determining tissue tropism and host specificity. S1 has been identified as a critical pro-apoptotic factor, regulating cell death through two distinct mechanisms. First, S1 activates caspase-8 and caspase-3, along with the cleavage of poly(ADP-ribose) polymerase (PARP), resulting in caspase-dependent apoptosis in Vero cells [33]. Second, S1 triggers the translocation of apoptosis-inducing factor (AIF) from mitochondria to the nucleus, resulting in caspase-independent apoptosis [31]. In addition, nsp5 (3C-like protease, 3CLpro), encoded in ORF1a, processes viral polyproteins into functional nonstructural proteins required for viral replication. Nsp5has been reported to disrupt mitochondrial membrane potential, promote cytochrome c release, and activate caspase-9/-3, ultimately leading to mitochondria-mediated intrinsic apoptosis [34].

Conversely, PEDV can suppress apoptosis through specific viral proteins to prolong host cell survival and enhance viral replication (Table 1). For instance, the accessory transmembrane protein (viroporin) ORF3has been proposed to inhibit apoptosis by blocking procaspase-3 activation, thereby facilitating viral replication [35]. Similarly, PEDV papain-like protease 2 (PLP2), endowed with deubiquitinase activity, cleaves viral polyproteins and antagonizes host innate immunity by suppressing type I interferon production. Mechanistically, PLP2 may stabilize MDM2 and promote p53 degradation, thus inhibiting p53-dependent apoptosis [36,37]. Furthermore, PEDV nsp9 is a nonstructural protein that functions as an RNA-binding protein, essential for viral RNA synthesis. Nsp9 has been shown to interact with the host factor histone cluster 2 H2BE (H2BE), suppressing endoplasmic reticulum stress (ERS)-induced apoptosis [38].

**Figure 1:**
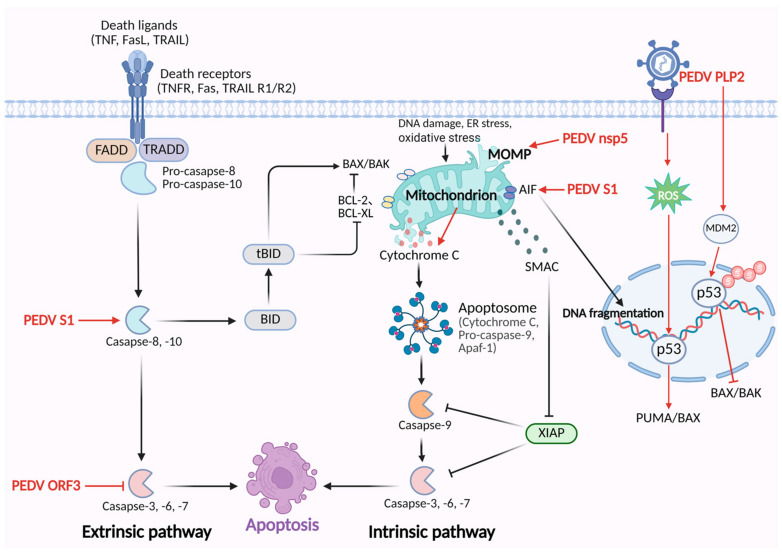
The mechanisms of PEDV regulating the apoptosis pathway. PEDV S1 protein activates caspase-8 and caspase-3 to trigger the extrinsic apoptosis. Additionally, PEDV S1 triggers the translocation of apoptosis-inducing factor (AIF) from mitochondria to the nucleus, leading to caspase-independent apoptosis. PEDV nsp5 disrupts mitochondrial membrane potential, resulting in the release of cytochrome c and activation of caspase-9/-3-induced apoptosis. PEDV ORF3 inhibits apoptosis through suppressing caspase-3 activation. PEDV PLP2 stabilizes MDM2 and promotes p53 degradation, inhibiting the transcription of pro-apoptotic BAX/BAK and then suppressing the apoptosis pathway. PEDV infection activates p53 by inducing the accumulation of ROS, which upregulates pro-apoptotic proteins, PUMA and Bax, and triggers intrinsic apoptosis.

Beyond these protein-specific mechanisms, accumulating evidence suggests that PEDV infection broadly modulates host signaling pathways to regulate apoptosis. Notably, PEDV infection induces excessive accumulation of reactive oxygen species (ROS), leading to p53 activation and nuclear translocation. Activated p53 subsequently upregulates pro-apoptotic proteins such as PUMA and Bax while downregulating the anti-apoptotic protein Bcl-2, thereby promoting mitochondria-mediated intrinsic apoptosis. Inhibition of ROS production or blockade of p53 activation markedly attenuates apoptosis and impairs PEDV replication [39,40]. The underlying mechanisms by which PEDV regulates cell apoptosis are summarized in Figure 1.

### 2.3. Apoptosis-Targeted Treatments in PEDV Infection

PEDV infection can trigger intrinsic and extrinsic apoptosis in host cells, suggesting that distinct therapeutic targets may be identified according to the specific pathway involved. Inhibition of intrinsic apoptosis primarily relies on preventing mitochondrial outer membrane permeabilization (MOMP) and cytochrome c release, focusing on preserving mitochondrial membrane integrity and regulating Bcl-2 family proteins. For instance, cyclosporine A can block the opening of the mitochondrial permeability transition pore (mPTP), stabilizing mitochondrial membrane potential [41,42]. Various antioxidants, such as N-acetylcysteine (NAC), Trolox, and Tempol, have been shown to suppress ROS-p53-mediated intrinsic apoptosis [43]. In addition, specific caspase-9 inhibitors, such as Z-LEHD-FMK, can directly interrupt the caspase cascade initiated by apoptosome activation [44].

In contrast, strategies to suppress extrinsic apoptosis involve blocking death receptor (DR) signaling and caspase activation, with caspase inhibition representing one of the most promising approaches. The irreversible pan-caspase inhibitor Z-VAD-FMK has been reported to prevent apoptosis induced by multiple viruses, including Japanese encephalitis virus, HIV, and hepatitis viruses [44]. Another pan-caspase inhibitor, Emricasan, has been shown to block human cortical neural progenitor cells apoptosis during Zika infection [45]. Similarly, apoptosis in Venezuelan equine encephalitis virus infection, is mediated by death receptor pathway activation, and Z-IETD-FMK can specifically inhibit caspase-8 activity to preserve neuronal survival [44]. Collectively, these findings highlight the potential therapeutic value of caspase inhibitors in treating virus-induced apoptosis. Nevertheless, inhibiting cell death pathways may also interfere with beneficial antiviral immune responses, emphasizing the need to carefully balance antiviral efficacy and host defense.

Therapeutic research against PEDV has explored various inhibitors and natural compounds targeting viral proteins, although most candidates remain at the in vitro or preclinical stage. Targeting the viral 3C-like protease (nsp5) represents a major antiviral strategy. Several natural compounds, including baicalein, baicalin, and quercetin [46,47], as well as small-molecule inhibitors such as GC376 [48], have been reported to inhibit PEDV 3CLpro and suppress viral replication. This antiviral effect may be partly mediated through the alleviation of nsp5-induced mitochondrial dysfunction and apoptosis.

## 3. PEDV Infection and Necroptosis

### 3.1. Necroptosis Machinery

Necroptosis, first defined in 2005 by J. Yuan, is a form of programmed cell death (PCD) distinct from apoptosis [49]. This process primarily depends on receptor-interacting protein kinase 1 (RIPK1), receptor-interacting protein kinase 3 (RIPK3), and mixed lineage kinase domain-like pseudokinase (MLKL). Necroptosis is characterized by plasma membrane rupture, organelle swelling, leakage of intracellular contents, and the release of damage-associated molecular patterns (DAMPs) [50]. Accumulating evidence indicates that necroptosis contributes to the initiation and progression of a wide range of pathological conditions, including inflammatory diseases [51], neurodegenerative disorders [52], pulmonary diseases [53], liver-associated pathologies [54,55], and cardiovascular diseases [56].

Extracellular ligands binding to their cognate cell surface receptors can initiate necroptosis. Among these, death-receptor-mediated necroptosis has been studied extensively, with the TNFR1 signaling pathway as the classical model. Upon binding of TNF-α to its receptor TNFR1, the death domain (DD) of TNFR1 recruits the adaptor protein TRADD, which in turn engages RIPK1 and TRAF2, facilitates the involvement of the E3 ubiquitin ligases cIAP1 and cIAP2 [57], and recruits the linear ubiquitin chain assembly complex (LUBAC) [58], together forming complex I. Under physiological conditions, complex I promotes cell survival and inflammatory responses by recruiting TAK1 and the IKK complex, activating the NF-κB and MAPK signaling pathways [59,60]. When cIAP1/2 are depleted or TAK1 kinase is inhibited, RIPK1 undergoes deubiquitination, and complex I transitions into complex II, composed of FADD, TRADD, and caspase-8, which activates the caspase-8–mediated apoptotic pathway [61,62,63]. In the absence of caspase-8 activity, however, apoptosis is diverted to necroptosis. Loss of caspase-8 function abrogates its cleavage of RIPK1 and RIPK3, thereby promoting their phosphorylation [64,65,66]. RIPK1 interacts with RIPK3 through their RHIM domains to form the necrosome, which subsequently activates the downstream effector MLKL. Activated MLKL undergoes oligomerization and inserts into the plasma membrane to form pores [67,68,69], leading to the release of cellular-damage-associated molecular patterns (cDAMPs) such as mitochondrial DNA (mtDNA), high-mobility group box 1 (HMGB1), IL-1β, and IL-33, along with proinflammatory cytokines, thus driving inflammation and eliciting immune responses [70,71]. In addition, FasL and TRAIL can also trigger necroptosis by binding to their respective death receptors, Fas and DR4/5, recruiting FADD and caspase-8 to form the death-inducing signaling complex (DISC) [72,73].

In addition, lipopolysaccharide (LPS) and viral double-stranded RNA (dsRNA) can activate Toll-like receptors (TLRs) located on the plasma membrane, leading to recruitment of the adaptor protein TRIF. This signaling event can induce phosphorylation of RIPK3 and MLKL in an either RIPK1-dependent or -independent manner, thus driving necroptosis [74,75]. Moreover, Z-DNA binding protein 1 (ZBP1) has been identified as a critical nucleic acid sensor. Through its Zα domain, ZBP1 recognizes viral Z-form nucleic acids (Z-NA) or endogenous RNA and subsequently recruits RIPK3 via its RHIM domain, resulting in MLKL phosphorylation and the initiation of necroptosis [76,77,78,79]. This ZBP1-mediated pathway represents an important and increasingly recognized mechanism for regulating necroptosis that warrants further investigation.

### 3.2. Necroptosis Regulation in PEDV Infection and Targeted Treatments

**Figure 2:**
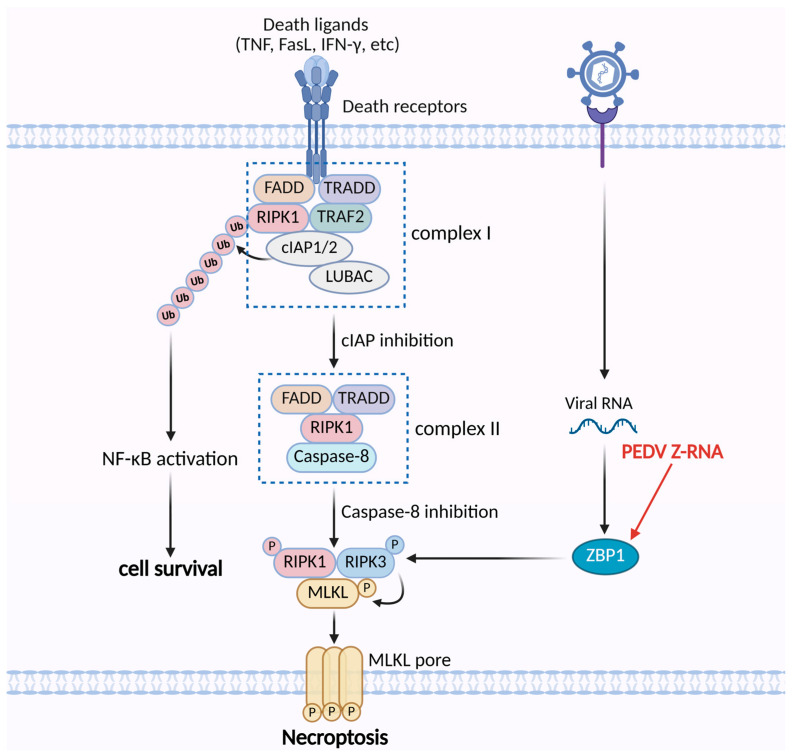
The mechanisms of PEDV regulating the necroptosis pathway. PEDV Z-RNA can be sensed by ZBP1, which activates RIPK3 to trigger necroptosis.

Cell death induced by porcine epidemic diarrhea virus (PEDV) has traditionally been studied primarily in the context of apoptosis. Recent findings, however, have demonstrated that necroptosis also plays a role in PEDV infection. Both in vivo studies in infected piglets and in vitro experiments in Vero E6 cells revealed upregulation of necroptosis-associated marker proteins and co-localization of Z-NA and ZBP1 in infected cells. Silencing or knockout of ZBP1 significantly reduced necroptotic activity, confirming that PEDV infection triggers ZBP1-mediated necroptosis [80]. As no previous reports have addressed this mechanism, many questions remain to be investigated. Insights from studies of other coronaviruses may help infer the precise regulatory processes underlying PEDV-induced necroptosis. In SARS-CoV-2 infection, Z-RNA generation activates ZBP1-RIPK3-MLKL–mediated necroptosis and inflammatory responses, a process that, unlike TNFR1-mediated necroptosis, proceeds independently of RIPK1 [81]. Moreover, SARS-CoV-2 nonstructural protein ORF3a, through its viroporin activity, disrupts host ion homeostasis, thereby activating necroptotic and apoptotic signaling pathways, ultimately promoting cell death and exacerbating COVID-19 pathogenesis [82]. The SARS-CoV-2 envelope (E) protein has been shown to trigger necroptosis and promote inflammatory responses in lung and colon epithelial cells through the activation of the RIPK1 signaling pathway [83]. Taken together, these observations from other coronaviruses provide a valuable framework for hypothesizing how PEDV may trigger similar necroptotic processes. Further investigation of PEDV viral protein functions and RNA structures may reveal whether such mechanisms are conserved across coronaviruses. The underlying mechanisms by which PEDV regulates necroptosis are summarized in Figure 2.

Host cells employ necroptosis as a defense mechanism to restrict viral replication; however, this process also results in plasma membrane rupture, the release of intracellular contents, and subsequent inflammatory responses. Targeting key mediators of the necroptotic pathway, such as RIPK1, RIPK3, and MLKL, therefore, represents a promising therapeutic strategy for porcine epidemic diarrhea (PED). Since clinical interventions specifically addressing PEDV-induced necroptosis are currently lacking, insights must be drawn from therapeutic approaches explored in the context of other viral infections. For instance, Necrostatin-1 and its derivative Nec-1s, both RIPK1 inhibitors, have been shown to attenuate RIPK1-dependent necroptotic signaling, thereby reducing inflammation and tissue injury caused by SARS-CoV-2 infection, as well as mitigating cell death and inflammatory responses in acute lung injury (ALI) [84,85,86,87]. Primidone, an approved drug, has also been demonstrated in vitro to suppress RIPK1-mediated necroptosis, suggesting therapeutic potential in COVID-19 patients [88]. In addition, RIPK3 inhibitors such as GSK’872, GSK’843, GSK’840, and UH15-38 have exhibited efficacy in multiple viral infection models [87,89]. Although these RIPK1- and RIPK3-targeting inhibitors have yet to be evaluated in the context of PED, and no experimental data are currently available, prior studies in other viral infections highlight their potential translational value as candidate strategies for PED management.

## 4. PEDV Infection and Pyroptosis

### 4.1. Pyroptosis Machinery

Pyroptosis is a type of programmed cell death triggered by inflammasome activation, characterized by DNA fragmentation, cellular swelling, membrane pore formation, and the release of proinflammatory factors [90]. This process depends on inflammatory caspases and the gasdermin (GSDM) family members (mainly Gasdermin A, B, C, D and E) [91]. Once activated, caspases cleave GSDMs, releasing the N-terminal effector domain from the C-terminal repressor domain. The liberated N-terminal fragment then binds to membrane lipids and forms pores, disrupting osmotic balance and ultimately causing membrane rupture [91,92,93,94]. Unlike apoptosis, pyroptosis elicits a strong inflammatory response and contributes to the development of many diseases, including autoimmune diseases, cancer, neurodegenerative disorders, inflammatory bowel disease, and COVID-19 [95,96,97,98].

Two major pathways regulate pyroptosis: the caspase-1–dependent canonical pathway and the caspase-4/5/11–dependent noncanonical pathway [93,96]. In the canonical pathway, inflammasomes are assembled from three parts: a sensor protein, the adaptor ASC, and the effector caspase-1 [99,100,101]. Sensor proteins include nucleotide-binding oligomerization-domain-like receptors (e.g., NLRP1, NLRP3, NLRP6, NLRC4, NAIP), AIM2-like receptors, and RIG-I–like receptors. Pattern-recognition receptors (PRRs), such as TLRs, TNFR, and IL-1R1, detect pathogen-associated molecular patterns (PAMPs) or damage-associated molecular patterns (DAMPs), activating the NF-κB pathway and upregulating the expression of NLRP3, pro–IL-1β, and pro–IL-18. Subsequent inflammasome assembly (e.g., NLRP3 or AIM2) activates caspase-1, which cleaves pro–IL-1β and pro–IL-18 into mature forms [102,103,104]. Additionally, caspase-1 cleaves GSDMD to release the N-terminal domain, which promotes ionic efflux and organelle leakage, while also enabling the secretion of IL-1β and IL-18, thereby amplifying inflammatory responses [93]. The noncanonical pathway is typically triggered by cytosolic lipopolysaccharide (LPS), which directly activates caspase-4 and caspase-5 in humans or caspase-11 in mice [105,106,107]. These caspases usually cleave GSDMD, leading to pore formation and pyroptotic cell death.

Emerging evidence suggests that caspase-3 and caspase-8 can convert apoptosis to pyroptosis under certain conditions. For instance, the Yersinia effector protein YopJ suppresses TAK1 or IKK kinases, leading to caspase-8-dependent cleavage of GSDMD [108,109]. It is reported that chemotherapy activates caspase-3 through the BAK/BAX-caspase-9 signaling pathway, which directly cleaves GSDME and then releases the pore-forming N-terminal fragment. In animal models, knockdown of GSDME attenuates tissue damage induced by chemotherapy [110,111,112].

### 4.2. Pyroptosis in PEDV Infection

Clinically, PEDV infection can lead to acute enteritis in piglets, which may be caused by pyroptosis and the release of inflammatory cytokines from intestinal epithelial cells. As reported in IPEC-J2 cells, PEDV causes mitochondrial dysfunction, leading to the generation of mitochondrial reactive oxygen species (mtROS) and the release of mitochondrial DNA (mtDNA). This process activates the NF-κB signaling pathway and NLRP3 inflammasome, where caspase-1 cleaves pro-IL-1β and GSDMD to facilitate the release of inflammatory cytokines (such as IL-1β). Similar processes have also been observed in Vero cells [113,114]. ZBP1 triggers ROS-mediated oxidative stress by recognizing Z-NA, produced during PEDV proliferation, thus inducing apoptosis, necroptosis, and pyroptosis both in vitro and in vivo [80]. Although PEDV has been shown to induce pyroptosis via ROS-mediated mitochondrial dysfunction and oxidative stress, the specific viral proteins and upstream signaling events remain unclear. Evidence from studies of other coronaviruses provides a valuable framework that PEDV may employ to regulate pyroptosis. For instance, the ORF3a protein of SARS-CoV and SARS-CoV-2 promotes inflammasome activation and pyroptosis via its viroporin activity [115]. These examples illustrate how specific viral proteins can modulate inflammasome signaling and pyroptosis, suggesting that PEDV may employ similar strategies to manipulate pyroptosis.

**Figure 3:**
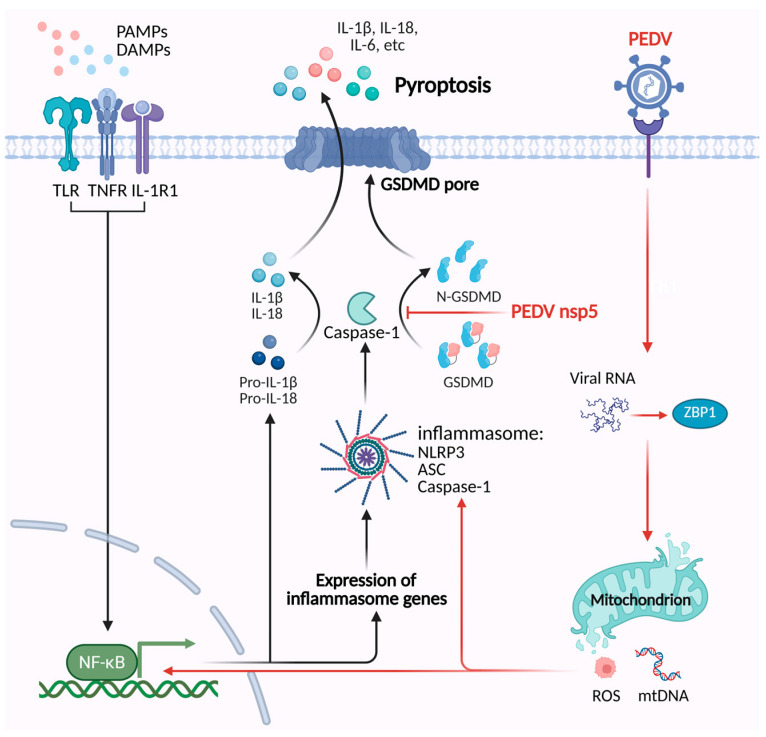
The mechanisms of PEDV regulating the pyroptosis pathway. PEDV Z-RNA can be recognized by ZBP1, which triggers ROS-mediated oxidative stress and the release of mtDNA. This process activates the NF-κB signaling pathway and NLRP3 inflammasome, which induces pyroptosis. PEDV nsp5 cleaves porcine GSDMD into two inactive fragments, which inhibit pyroptosis.

While moderate inflammation can help suppress viral replication through various downstream signaling pathways, excessive inflammation may be fatal to the host. Consequently, viruses have evolved diverse mechanisms to modulate host inflammation, balancing their replication needs with host survival. For instance, PEDV utilizes its nonstructural protein nsp5 to cleave porcine GSDMD (pGSDMD) into two inactive fragments derived from p30. These fragments cannot trigger pyroptosis, which ultimately facilitates viral replication in the early stages of infection [116]. Similar mechanisms have been observed in other coronaviruses. For example, the nucleocapsid protein of SARS-CoV-2 interacts with the linker region of GSDMD, preventing caspase-1 cleavage and inhibiting pyroptosis [117]. These findings suggest that avoiding pyroptosis may be a common strategy used by coronaviruses to maintain infection. The underlying mechanisms by which PEDV regulates pyroptosis are summarized in Figure 3.

### 4.3. Pyroptosis-Targeted Treatments in PEDV Infection

Pyroptosis induced by PEDV contributes to inflammatory responses and tissue damage. Therapeutic strategies targeting pyroptosis have the potential to alleviate virus-induced pathological changes. Inhibition of inflammasome assembly, particularly the NLRP3 inflammasome, has shown efficacy in suppressing pyroptosis. A notable inhibitor, MCC950, selectively binds to the NACHT domain of NLRP3, obstructing its ATPase activity and preventing inflammasome assembly [118]. Evidence indicates that MCC950 enhances survival rates in SARS-CoV-2–infected mice, reduces tissue inflammation, suppresses NLRP3 inflammasome activation induced by influenza A virus (IAV) in human bronchial epithelial cells, and improves outcomes in rat models of chronic obstructive pulmonary disease (COPD) [119,120,121]. Additionally, other agents such as CY-09, Dapansutrile (OLT1177), and Tranilast can block the assembly of the NLRP3 inflammasome [122,123,124,125]. ROS-mediated oxidative stress has been reported to activate the NLRP3 inflammasome, which leads to pyroptosis [126,127]. The antioxidant N-acetylcysteine (NAC) can lower ROS levels and suppress oxidative-stress-mediated pyroptosis in infections caused by PEDV and other coronaviruses, including SARS-CoV-2 [128,129].

In addition, VX-765 exerts protective effects by directly inhibiting caspase-1 activity, which prevents the maturation and secretion of IL-1β and IL-18, as well as the cleavage of GSDMD, ultimately reducing virus-induced pyroptosis and inflammation [130]. Similarly, water extracts of Portulaca oleracea (WEPO) and its active compound, quercetin, have been reported to suppress caspase-1 activation, alleviating inflammation and emerging as potential candidates for preventing or treating PEDV [113].

More recently, attention has shifted to inhibitors of GSDMD, known as the executioner of pyroptosis. Necrosulfonamide (NSA), a known MLKL inhibitor, could bind to Cys191/Cys192 (human/mouse) of GSDMD, thereby blocking their oligomerization, without influencing the cleavage or initial dimerization of GSDMD [131]. Moreover, NSA can also suppress pyroptosis by targeting the gene transcription of LPS and the activation of caspase-1 in other models [132,133]. Disulfiram, used to treat alcohol use disorder, has been reported to prevent GSDMD pore formation, which reduces IL-1β release and mortality in murine models infected by SARS-CoV-2 [132,134]. Dimethyl fumarate (DMF), utilized to therapeutically manage MS, has been shown to prevent caspase-1/GSDMD interaction rather than caspase-1 cleavage, finally inhibiting GSDMD-dependent pyroptosis. In addition, DMF can also succinate GSDME at the Cys45 site to block GSDME cleavage and GSDME-dependent pyroptosis [135].

Collectively, these compounds show significant therapeutic potential for reducing inflammation and inhibiting pyroptosis. However, further studies are needed to explore their safety and efficacy in the context of PEDV infections.

## 5. PEDV Infection and PANoptosis

### 5.1. PANoptosis Machinery

PANoptosis was first proposed by Kanneganti in 2019 as a novel form of programmed cell death characterized by the simultaneous activation of pyroptosis, apoptosis, and necroptosis (“P” for pyroptosis, “A” for apoptosis, and “N” for necroptosis) [136,137]. The execution of PANoptosis critically relies on the assembly of a multiprotein complex known as “PANoptosome”, which functions as a signaling hub linking diverse upstream stimuli to downstream death effector pathways [138]. Upon pathogenic challenge or cellular stress, pattern recognition receptors (PRRs) such as ZBP1, AIM2, and NLRP3 become activated and recruit essential adaptor proteins (e.g., ASC, FADD) and effector molecules (e.g., caspase-1/8, RIPK1, RIPK3), leading to the formation of the PANoptosome. This complex concurrently initiates multiple cell death pathways, including pyroptosis via caspase-1/GSDMD, apoptosis through caspase-8/caspase-3/7, and necroptosis mediated by RIPK3-MLKL [137,139,140,141]. Notably, recent evidence indicates that caspase-6 promotes PANoptosome assembly by enhancing the interaction between RIPK3 and ZBP1, suggesting that caspases traditionally associated with apoptosis may also exert scaffolding functions [142].

PANoptosis contributes to host defense against pathogenic invasion and, when excessively activated, to pronounced inflammatory responses and tissue injury. It has been implicated as a critical contributor to the pathogenesis of diverse conditions, including infectious diseases [137,143,144], autoimmune disorders [145], neurodegenerative diseases [146], cardiovascular diseases [147,148], and cancer [149,150]. Consequently, elucidating the regulatory mechanisms of PANoptosis and the assembly dynamics of the PANoptosome has emerged as a forefront focus in cell death research.

### 5.2. PANoptosis in PEDV Infection

**Figure 4:**
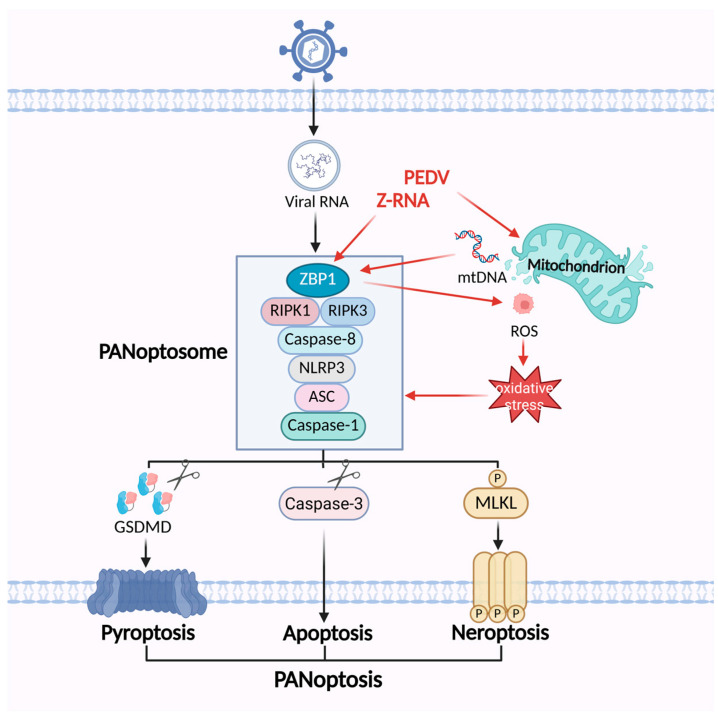
The mechanisms of PEDV regulating the PANoptosis pathway. PEDV infection generates Z-RNA and releases mtDNA, which can be sensed by ZBP1 and triggers the assembly of PANoptosome. ZBP1 promotes mitochondrial damage as well as ROS-mediated oxidative stress, which in turn facilitates the formation of the ZBP1-mediated PANoptosome.

PEDV infection generates Z-form RNA (Z-RNA), which is sensed by ZBP1, leading to ROS accumulation and oxidative stress activation. Thus, the process amplifies ZBP1-mediated cell death signaling, including pyroptosis via GSDMD, apoptosis via caspase-3, and necroptosis via MLKL, which eventually drives the execution of PANoptosis. While this process restricts viral replication, it simultaneously provokes severe inflammatory responses and disruption of the intestinal barrier, thereby aggravating disease pathology. These findings reveal a novel cell death mechanism underlying PEDV pathogenesis and provide critical insights into virus–host interactions as well as potential therapeutic targets [128]. The underlying mechanisms by which PEDV regulates PANoptosis are summarized in Figure 4.

## 6. Conclusions and Perspectives

In summary, the interaction between cell death and viral infection involves complex mechanisms. Host cells can limit viral replication and spread through various death pathways, serving as a defense strategy against viral invasion. However, excessive activation of these pathways can lead to accelerated viral dissemination, severe tissue damage, and exacerbated inflammatory responses, such as cytokine storms. PEDV has evolved multiple strategies to regulate cell death, including apoptosis, necroptosis, pyroptosis, and PANoptosis, thereby influencing the progression of PED. Nevertheless, the specific molecular mechanisms by which PEDV regulates cell death remain incompletely understood. In particular, upstream events in pathways like necroptosis and pyroptosis are still unclear, even though their occurrence has been confirmed. Beyond the incomplete understanding of cell death pathways, the relative importance of each pathway and their temporal sequence during infection remains unclear. Current studies are limited in addressing whether one pathway predominates at early versus late stages of infection, or how these pathways may cross-regulate each other in the context of PANoptosis. Further experimental studies are needed to elucidate the dynamics and interactions among PEDV-induced cell death pathways.

Future studies should focus on identifying the signaling pathways through which PEDV activates necroptosis, pyroptosis, and other forms of cell death. Elucidating the mechanisms underlying Z-RNA formation, along with the molecular determinants that allow its recognition by host sensors, will offer critical insights into virus–host interactions and the initiation of cell death signaling pathways. In parallel, exploring the crosstalk between the ZBP1-mediated PANoptosome and other innate immune sensors during PEDV infection is essential to understand how multiple signaling pathways integrate to regulate apoptosis, necroptosis, and pyroptosis. Such studies may reveal potential checkpoints or synergistic targets for therapeutic intervention and clarify how PEDV manipulates host immune responses to favor viral replication while avoiding excessive immunopathology.

To date, several inhibitors and drugs targeting cell death pathways have been developed for antiviral therapy. Some have received clinical approval, while others are still undergoing trials. However, several candidates have failed due to limited efficacy or off-target effects. Notably, there is a lack of clinical data regarding the use of these inhibitors in treating PEDV, and further trials are necessary to evaluate their efficacy. Since PEDV infection activates multiple cell death pathways, inhibiting just one pathway may result in compensatory activation of alternative pathways. Therefore, combination strategies that target multiple death pathways simultaneously may be essential, requiring careful selection and coordination of different inhibitors or drugs. For example, preclinical evaluation of combined therapies, such as an NLRP3 inhibitor paired with an antioxidant, in animal models of PED, could provide valuable insights into therapeutic efficacy and safety. Such approaches may lead to safer and more effective treatments, reducing the damage and economic losses caused by porcine epidemic diarrhea virus.

## Figures and Tables

**Table 1:** Mechanisms of PEDV viral proteins in modulating apoptosis.

Viral Protein	Mechanism	Effect on Apoptosis	References
S1	Activates caspase-8/-3, induces PARP cleavage	Promotes caspase-dependent apoptosis	[33]
S1	Triggers translocation of AIF from the mitochondria to the nucleus	Promotes caspase-independent apoptosis	[31]
nsp5	Causes mitochondrial dysfunction and cytochrome c release, activates caspase-9/-3	Facilitates intrinsic apoptosis	[34]
ORF3	Inhibits activation of procaspase-3	Inhibits apoptosis	[35]
PLP2	Stabilizes MDM2, promotes p53 degradation	Suppresses apoptosis	[36,37]
nsp9	Interacts with histone cluster 2 H2BE	Suppresses ER-stress-induced apoptosis	[38]

## Data Availability

No new data were created or analyzed in this study. Data sharing is not applicable to this article.
